# Invited commentary: “Risk of neuropsychiatric disorders in primary hyperparathyroidism: Parathyroidectomy versus nonoperative management”

**DOI:** 10.1002/wjs.12318

**Published:** 2024-08-21

**Authors:** Christine J. O’Neill

**Affiliations:** ^1^ John Hunter Hospital and University of Newcastle New Lambton Heights New South Wales Australia

Surgical treatment for primary hyperparathyroidism (PHPT) has traditionally prioritized “bones and stones” over “moans and groans”. As widespread routine biochemical testing and screening for osteoporosis becomes commonplace, more patients with “asymptomatic” or “minimally symptomatic” disease are being diagnosed. Strong indications for surgery remain confined to young patients and those with established osteoporosis or renal calculi.^1^ There remains some reluctance to refer patients for surgery where disease is “asymptomatic” particularly in those aged >50 years.^2^ Current guidelines focus on biochemical cure and longer‐term improvements in bone mineral density and reduction in renal calculi as measures of success following surgery.^1^

From the patient perspective, the most concerning symptoms are often the “nonspecific symptoms” of PHPT. These include anxiety, depression, fatigue, cognitive decline, aches, and pains. Improvements in these “nonspecific symptoms” are well‐documented following surgery and can be measured by both validated disease‐specific patient‐reported outcome measures (PROMs) and correlated with global health‐related quality of life scores.^3^ As clinicians, it is difficult to predict the degree to which any individual patient will benefit from surgery particularly regarding neurocognitive symptoms. Why one patient bounds back into the clinic after surgery and another is disappointed at the lack of improvement in symptoms is not fully understood. Longer‐term data on neurocognitive symptom change and the development of neuropsychiatric diagnoses after parathyroidectomy is lacking.

In this manuscript, Song and colleagues investigate the effect of parathyroidectomy on the subsequent diagnosis of neuropsychiatric disorders.^4^ The study population is a large retrospective cohort of 3728 patients from their institutional database, encompassing health data from a population of over one million individuals. Notably, individuals with both “classical PHPT” (with elevation of both serum calcium and PTH) and “normohormonal PHPT” (elevation of serum calcium with normal PTH) are included. Normocalcemic patients and those with secondary causes of hyperparathyroidism are excluded. Just under half of the patients received surgery.

Baseline differences in age, race, marital status, and PTH levels between the nonoperative and operative groups suggest some bias in surgical selection. Propensity score matching has been used to adjust for these potential confounders. At a median of 4.2 years follow‐up, there was a significantly lower rate of new diagnoses of cognitive impairment, somnolence, and schizophrenia in the operative group. Diagnoses of anxiety, depression, and suicidal ideation were not influenced by operative management. In addition, a decreased likelihood of needing future hospitalization for neuropsychiatric disorders was found in those who underwent parathyroidectomy.

The authors are to be congratulated on putting together such a large dataset investigating this important question. Steps have been taken to ensure the completeness of the data; reporting only a 3.4% loss to follow‐up. The authors have been open about the limitations of their research and measured in their conclusions. Unlike prior studies on this topic that have utilized PROMs, the outcome measure in this study involves a neuropsychiatric diagnosis. No threshold for “diagnosis” is given, but it is probable that only those who sought health care for neuropsychiatric symptoms severe enough to warrant a “diagnosis” are included in this study. Thus, although the subtleties of the patient experience (including less prominent anxiety and depressive symptoms) are unlikely to be captured, this study brings us data about healthcare utilization for neuropsychiatric symptoms including hospitalization. The suggestion that parathyroidectomy might prevent or delay future neuropsychiatric disorders is persuasive. Further prospective data are required to confirm this association, establish causation, and identify those subsets of patients who are most likely to benefit.

Nevertheless, this study brings to the evidence base tantalizing data indicating a possible role in neuropsychiatric health prevention (or preservation) with parathyroidectomy. This is likely to be an attractive proposition to many patients and their physicians and suggests that the indications for parathyroidectomy in “asymptomatic PHPT” be expanded. This is consistent with evolving modern surgical practice, including the recent Australian and New Zealand guidelines advocating for surgery in any patient with PHPT and an expected life expectancy of >10 years.^5^

For many years, endocrine surgeons have argued that “asymptomatic PHPT” is not truly asymptomatic and that surgery should be considered in all patients fit for anesthesia. This manuscript suggests that we move a little further beyond this notion, and turn our focus to the role of parathyroidectomy in preventing future health morbidity (physical and psychological), to the longer term benefit of both our patients and the health system.

## AUTHOR CONTRIBUTIONS

**Christine J. O'Neill**: Conceptualization; writing—original draft; writing—review & editing.
